# Global burden of maternal and congenital syphilis and associated adverse birth outcomes—Estimates for 2016 and progress since 2012

**DOI:** 10.1371/journal.pone.0211720

**Published:** 2019-02-27

**Authors:** Eline L. Korenromp, Jane Rowley, Monica Alonso, Maeve B. Mello, N. Saman Wijesooriya, S. Guy Mahiané, Naoko Ishikawa, Linh-Vi Le, Morkor Newman-Owiredu, Nico Nagelkerke, Lori Newman, Mary Kamb, Nathalie Broutet, Melanie M. Taylor

**Affiliations:** 1 Avenir Health, Geneva, Switzerland; 2 Independent consultant, London, United Kingdom; 3 Department of Communicable Diseases and Environmental Determinants of Health, Pan-American Health Organization, Washington DC, United States of America; 4 Independent Consultant, Atlanta, Georgia, United States of America; 5 Avenir Health, Glastonbury, Connecticut, United States of America; 6 World Health Organization, Regional Office for the Western Pacific, Manila, the Philippines; 7 World Health Organization, Sub-Saharan Africa Office, Brazzaville, Republic of Congo; 8 Independent Consultant, Leiden, The Netherlands; 9 USA Centers for Disease Control and Prevention, Cambodia Country Office, Phnom Penh, Cambodia; 10 USA Centers for Disease Control and Prevention, Division of STD Prevention, Atlanta, Georgia, United States of America; 11 World Health Organization, Dept. of Reproductive Health and Research, Geneva, Switzerland; BITS Pilani, INDIA

## Abstract

**Background:**

In 2007 the World Health Organization (WHO) launched the global initiative to eliminate mother-to-child transmission of syphilis (congenital syphilis, or CS). To assess progress towards the goal of <50 CS cases per 100,000 live births, we generated regional and global estimates of maternal and congenital syphilis for 2016 and updated the 2012 estimates.

**Methods:**

Maternal syphilis estimates were generated using the Spectrum-STI model, fitted to sentinel surveys and routine testing of pregnant women during antenatal care (ANC) and other representative population data. Global and regional estimates of CS used the same approach as previous WHO estimates.

**Results:**

The estimated global maternal syphilis prevalence in 2016 was 0.69% (95% confidence interval: 0.57–0.81%) resulting in a global CS rate of 473 (385–561) per 100,000 live births and 661,000 (538,000–784,000) total CS cases, including 355,000 (290,000–419,000) adverse birth outcomes (ABO) and 306,000 (249,000–363,000) non-clinical CS cases (infants without clinical signs born to un-treated mothers). The ABOs included 143,000 early fetal deaths and stillbirths, 61,000 neonatal deaths, 41,000 preterm or low-birth weight births, and 109,000 infants with clinical CS. Of these ABOs– 203,000 (57%) occurred in pregnant women attending ANC but not screened for syphilis; 74,000 (21%) in mothers not enrolled in ANC, 55,000 (16%) in mothers screened but not treated, and 23,000 (6%) in mothers enrolled, screened and treated. The revised 2012 estimates were 0.70% (95% CI: 0.63–0.77%) maternal prevalence, and 748,000 CS cases (539 per 100,000 live births) including 397,000 (361,000–432,000) ABOs. The estimated decrease in CS case rates between 2012 and 2016 reflected increased access to ANC and to syphilis screening and treatment.

**Conclusions:**

Congenital syphilis decreased worldwide between 2012 and 2016, although maternal prevalence was stable. Achieving global CS elimination, however, will require improving access to early syphilis screening and treatment in ANC, clinically monitoring all women diagnosed with syphilis and their infants, improving partner management, and reducing syphilis prevalence in the general population by expanding testing, treatment and partner referral beyond ANC.

## Introduction

Mother-to-child transmission (MTCT) of syphilis during pregnancy can lead to serious fetal outcomes in the second or third trimester including early fetal death, stillbirth, neonatal death, preterm birth, low birthweight and congenital infection in infants [1]. Syphilis is the second most common infectious cause of stillbirth worldwide [2] and an important preventable contributor to infant morbidity and mortality [1].

Preventing MTCT of syphilis through expanded early testing in antenatal care (ANC) and immediate treatment with a single injection of benzathine penicillin [3,4] is highly cost-effective [5,6], and eliminating MTCT of syphilis is feasible in settings where maternal prevalence is low enough.

In 2007 the World Health Organization (WHO) and partners launched the global initiative for the elimination of MTCT (EMTCT) of syphilis and HIV; the WHO Pan-American Health Organization and the WHO Western Pacific Region adopted regional resolutions for triple EMTCT of HIV, hepatitis B and syphilis in 2010 and 2017 [7], respectively. In 2014 WHO developed global guidance and criteria for the validation of EMTCT of HIV and syphilis: country validation of EMTCT of syphilis requires a congenital syphilis (CS) case rate of 50 or fewer cases per 100,000 live births and three process targets: (i) at least 95% coverage of ANC with at least one visit (ANC1) attained; (ii) at least 95% of pregnant women in ANC tested for syphilis; and (iii) at least 95% of women tested positive for syphilis received adequate treatment [8]. As of 1 June 2018, ten countries have been validated as having eliminated MTCT of syphilis [9–11].

The WHO Global Health Sector Strategy on Sexually Transmitted Infections 2016–2021 [12] identified as one of its four key targets for 2030 the elimination of CS as a public health problem, defined as ≤50 cases of CS per 100 000 live births, in 80% of countries. The following WHO case definition for CS applies to both national and global CS targets [13]:

- a stillbirth, live birth, or fetal loss at >20 weeks of gestation or >500 grams birthweight born to a syphilis seropositive mother without adequate syphilis treatment; OR
- a stillbirth, live birth, or child aged <2 years born to a woman with positive syphilis serology or unknown sero-status, and with laboratory and/or radiologic and /or clinical evidence of syphilis infection (regardless of the timing or adequacy of maternal treatment).

This definition includes both adverse pregnancy outcomes alternatively denoted as adverse birth outcomes (ABOs) and pregnant women who were not or inadequately treated, irrespective of birth outcome.

According to WHO, the estimated number of pregnant women with probable active syphilis (i.e., current infection likely able to be transmitted from mother-to-child) fell from 1.36 million in 2008 [14](revised to 1.49 million when the 2012 estimates were produced) to 930,000 in 2012 [15]; the number of ABOs fell from 520,905 in 2008 [14] (revised to 577,000 ABOs) to 351,000 in 2012 [15].

The objective of this analysis was to generate global and regional estimates of maternal and congenital syphilis for 2016 and 2012, and to assess progress in global EMTCT of syphilis. Output included prevalence of syphilis in pregnant women, number of pregnant women with syphilis, and resulting ABOs. In addition, we calculated the total number of CS cases including non-ABO cases, an indicator not produced in previous estimates. Results are presented as global and regional estimates. We have not presented country results, as the revised country input parameters and country level results are working estimates and not official national estimates.

## Methods

We used an approach similar to previous WHO estimations [15,16]. For each country, the maternal syphilis prevalence estimate was multiplied with annual number of pregnancies to estimate the number of women with probable active syphilis. This was combined with country data on ANC attendance, ANC syphilis screening coverage, and syphilis treatment coverage to generate numbers of treated and untreated syphilis infected pregnant women. We then applied relative risk probabilities to the number of treated and untreated infected women, to estimate numbers of CS cases and ABOs.

The new estimates differ from the old estimates in how the key country parameters (maternal syphilis prevalence, ANC1 coverage, ANC-based syphilis screening coverage, and ANC-based syphilis treatment coverage) were estimated. For the 2008 and 2012 estimates these inputs were based on the data reported by countries to the Global AIDS Monitoring System (GAM), and for those countries where no data were available a regional mean was used (imputed). Not all countries, however, reported through the GAM system and there were inconsistencies in the data. For the new estimates, the Spectrum-STI global database and Spectrum-STI model were used to generate national maternal syphilis estimates over time [17], and for the three service coverage data points the GAM reported data were supplemented with country data from other sources. Maternal syphilis trends were generated for each country over 2012–2016 drawing on data from the past decade rather than a single year. In addition, we refined the estimation of ABO risks in treated women to reflect the importance of treatment early during the pregnancy: the risk probability of ABOs for women with treated maternal syphilis was based on the country-specific average timing of first ANC visit, which was used as a proxy for ANC-based syphilis screening and treatment, rather than assuming the risk of ABO in treated women to be a fixed global value.

### Annual numbers of live births and pregnancies

Annual number of live births were based on the annual live births projection (2017 World Population Prospects, 2015–2020 estimates and 2015–2020 projections in the medium (i.e. intermediate-growth) variant [18]), adding to these country-specific annual stillbirths occurring from 28 or more weeks of gestation in each year within 2011 to 2015 previously estimated for 195 WHO member states [19]. For 2016 we used each country’s 2015 stillbirths estimate. For non-WHO member states, we assumed stillbirths to be 1.9% of live births, the median value over 2012–2015 across WHO member states [19].

### Maternal syphilis prevalence

Country estimates of maternal syphilis prevalence were based on data extracted from the Spectrum-STI adult prevalence database [20–22] developed by Avenir Health with support from WHO. The database synthesizes earlier multi-country databases and country data reported to the WHO, UNAIDS and UNICEF, through GAM. The vast majority of adult prevalence data comes from sentinel surveys and routine testing in pregnant women in ANC. In addition, there are a few data points from community studies and surveys among non-ANC (pregnant and/or non-pregnant) women, or adult men that were thought to be representative of the general adult population in the country [17,23]. For a subset of countries data from blood donor screening and published reports [24–34] were also included. When information was stratified by type of donor, we used the data from first-time donors only, since repeat donors may have a relatively lower syphilis risk, having already been screened for not having had blood-transmittable infections.

Prevalence data were standardized before trend fitting to ensure that they reflected active syphilis, defined as concurrent positivity on both a non-treponemal (e.g., Rapid Plasma Reagin (RPR) or Venereal Disease Research Laboratory (VDRL) test) and a treponemal test [35], as in previous WHO estimations [15,36]. Each data point was assigned a weight according to its national coverage and representativeness, and we assumed no systematic prevalence differences between the data types included [17].

Maternal syphilis prevalence was estimated using one of five approaches, depending on data availability:

A Countries with at least one study either in ANC women (sentinel survey or routine syphilis screening in ANC, with a sample size of >100) or in the general population from 2011 or later AND 3 or more syphilis prevalence measurements in ANC women or general adult populations from 2000 and 2017: Spectrum-STI model was used to generate a prevalence trend estimate for pregnant women. Data were fitted with smoothed-splines polynomial regression [17], using second order segmented polynomials. Both the number and positions of the knots were estimated, and the Akaike Information Criterion was used for model selection [17].
B Countries with one or two ANC data points from 2011 or later but fewer than three data points from 2000 to 2017: the average of the available data within 2011–2017 was used as the national estimate and assumed to be time constant for 2011–2016.
C Countries with no ANC or general population data from 2011–2017 but with one or more syphilis blood donor screening data from 2011 or later, and where including the post-2010 blood donor data meant that there were three or more measurements over 2000–2017: Spectrum-STI model used to generate a prevalence trend estimate, pooling pre-2011 ANC and general population data with the 2011–2017 blood donor data [17].
D Countries with no ANC or general population data from 2011–2017, and one or two blood donor screening data points from 2011–2017 and where even after including the post-2010 blood donor data there were fewer than three data points from 2000: a simple average of the prevalence in blood donors in any years reported within 2011–2017 was used as the national estimate and assumed to be time constant.
E Countries with no ANC, general population or blood donor data from 2011 or later: the value was the imputed median prevalence for all of the countries in the same WHO region that had an estimate based on national data—changing over time as the WHO regional median.

For countries where Spectrum-STI was used to generate maternal estimates (A and C) 95% confidence intervals (CI) were generated using the bootstrap resampling method, with methods and results described in detail before [17,20]. For each country, 400 samples were drawn and, at each iteration, both surveys’ observed prevalence and key parameters were re-sampled. The CIs obtained in this manner reflect stochastic variations in data based on sample sizes, uncertainties in diagnostic test adjustments, and the statistical model. For the other countries we assumed a 95% CI ranging from 50% of the point estimate as lower-bound, to 100%+75% of the point estimate as upper-bound, which was the median width of the 95% CIs across countries in group A.

### Service coverage

Based on data from a variety of sources covering the period 2008 to 2016, we developed a database of the three key service indicators: national ANC1 coverage, the proportion of pregnant women attending ANC screened for syphilis, and the proportion of pregnant women diagnosed with syphilis during ANC who were adequately treated. Data sources included: GAM data sets and other national programmatic and surveillance data sent in response to GAM data cleaning, national survey results, data provided during Spectrum-STI country workshops [35–38], and other published and unpublished national program reports, and regional WHO reports [10,11]. Country data review was conducted in collaboration with WHO regional STI advisors to complete the country coverage database and validate final data used.

National ANC1 coverage estimates were based on national reproductive and health surveys, collated in the DHS STAT compiler (https://www.statcompiler.com/en/) and the UNICEF global database (https://data.unicef.org/topic/maternal-health/antenatal-care/).

For all three indicators national estimates for each year between 2012 and 2016 were based on all of the identified data. Country data were reviewed by two of the authors (ELK and JR) for coherence across the indicators (e.g., number of women treated was smaller than the number diagnosed during ANC) and over time. Data provided from the WHO regional advisors or directly from countries and data from CS elimination reports took priority over the GAM data, which in turn took priority over other data sets.

For all three indicators we assigned the identified data to the year it was collected and then estimated the missing years using the closest year with data, with later years taking precedence over earlier years. For those countries with no eligible data point, an estimate was used (imputed). For high income countries, we used the mean value for each year of the high-income countries with data. For non-high-income countries, WHO region specific averages from countries with data were used. The regional means were generated for each year based on those countries with data, with each country weighted equally.

95% CIs on national coverages were calculated as (100%) ±25% of the proportion not ANC-covered, and (100%) ±50% of the proportions of pregnant women not attending ANC screened for syphilis, and those women diagnosed with syphilis during ANC but not adequately treated. ANC1 attendance and syphilis screening coverage in ANC were assumed to be independent of a woman’s syphilis status.

### Congenital syphilis and adverse birth outcomes

The WHO CS definition [13] was used to estimate total CS cases. This definition includes all syphilis-related ABOs in infants born to treated and untreated women as well as infants born alive, without clinical signs, to untreated syphilis-infected women (termed non-clinical cases). The risk probabilities of the various ABOs in untreated syphilis-infected mothers were assumed to be the same as in the previous WHO estimates and were based on a meta-analysis of various types of birth outcomes of pregnant women with syphilis. For pregnant women with untreated syphilis the risk probability of an ABO was 52%. ABOs included early fetal deaths or stillbirths (21%), preterm (born alive before 37 weeks’ gestation) or low birthweight (weighing <2500g; 6%), neonatal death (death in first 28 days; 9%), and clinical disease in infants (29–365 days; 16%) [1].

For women whose syphilis infection was adequately treated during pregnancy (defined as at least one dose of 2.4 million units of benzathine penicillin [4]), the WHO’s 2008 and 2012 estimates assumed that treatment reduced early fetal deaths or stillbirths by 82%, preterm or low birthweights by 65%, neonatal deaths by 80%, and clinical disease in infants by 97% [37]. This resulted in an estimated total risk of any syphilis-attributable ABO among treated mothers of 8% [14,15], fixed across countries. In the current estimates, the risk of developing an ABO in treated women was refined to take into account country-specific average timing of ANC enrollment, used as a proxy for ANC-based syphilis screening and treatment [38,39], implying that a country with early ANC enrolment has lower ABO rates than a country with late ANC enrolment. For countries with data on the proportion of pregnancies each year with early ANC attendance this was translated into an average time for first ANC visit and used as a proxy for the time of syphilis treatment among mothers diagnosed during ANC (S1 File). For countries without survey data, the timing of ANC1 was extrapolated and a time-varying ABO risk function in line with recent meta-analyses of ABO risk variation by pregnancy trimester [38,39] was applied to obtain a distribution of ABO risks across countries, with regional medians of 6.8% (range, 3.7–10.4%) in the African Region, 4.4% (2.7–6.8%) in the Region of the Americas, 5.5% (3.5–12.5%) in the Eastern Mediterranean Region, 3.4% (2.3–6.0%) in the European Region, 6.0% (2.9–7.9%) in the South-East Asia Region and 4.7% (2.3–12.2%) in the Western Pacific Region (S1 File).

Confidence intervals (95% CIs) for year-specific, national CS and ABO point estimates were generated by combining the variances of the key country-level input data—Maternal syphilis prevalence, ANC1 coverage, testing coverage and treatment coverage—and assuming that 95% CIs on the ABO probabilities were ±25% of the point estimate for both untreated and for treated mothers within a country. Uncertainties in annual numbers of pregnancies and live births were not considered. When generating regional and global uncertainty bounds we assumed the uncertainties were independent across countries. For translating uncertainties in individual parameters into uncertainties in outcome values we used the delta method (‘error formula method’) i.e. we linearized the formula for the outcome around the best value for the individual parameters and then applied the standard method of calculating variances of linear combinations of (assumed mutually uncorrelated) variables.

### Time trend evaluation

The absolute difference between 2012 and 2016 estimates was used to indicate the 5-year time trend. We were not able to determine p-values of trends in CS outcome, owing to a lack of data on how uncertainties in coverage parameters correlate across countries and across years. Making the assumption of independence across years did not appear to be realistic. For example, countries overreporting ANC attendance rates in one year are likely to do the same in another year. Information about relevant correlations was hard to come by unfortunately. We expect trends of sufficient magnitude to be of public health relevance, however.

### Ethics statement

No individual patient records were used in this study; we exclusively used aggregated data about numbers of patients, without any individual-patient information. All of these data had already been published or officially reported before, by national STI and/or maternal and child health programs and/or in scientific publications, this was all secondary material. Hence, no ethics review or approval was applicable

## Results

### Maternal syphilis prevalence

The Spectrum syphilis database as of 24 August 2018 contained over 1,400 data points collected post-1990 from 186 countries [17].

Countries were stratified into five groups based on the type and amount of data available (S2 File). Of all 205 countries, 134 had one or more ANC or general population data points from 2011 or later. Of these, 133 had 3 or more data points post-2000, and Spectrum-STI was used to generate maternal prevalence trends (group A). For one country the average of its available 2011–2017 ANC-based data points was used (B). Another 46 countries had no ANC data from 2011 or later and blood donor data was included in the analysis. Of these, 34 countries had sufficient data for a national Spectrum-STI trend estimate (C), and for 12 countries we used the average prevalence from their eligible 2011–2017 data points (D). For another 24 countries with no data, apart from the USA, prevalence trends were imputed as the regional estimate (E). For the USA, we used data provided by the US Centers for Disease Control and Prevention; maternal prevalence was assumed to be 0.14% based on the 0.17% adult prevalence of active syphilis measured in national household surveys over 2001–2004 [40], and held constant over 2012 to 2016.

The estimated global prevalence of maternal syphilis was 0.70% (95% confidence interval: 0.63–0.77%) in 2012 and 0.69% (0.57–0.81%) in 2016 (Table 1). Prevalence was also stable within most regions, except for non-significant increases in the Region of the Americas from 0.64% (0.56–0.71%) to 0.86% (0.76–1.03%) and in the Region of the Eastern Mediterranean from 0.69% (0.57–0.82%) to 0.77% (0.46–1.07%), and a non-significant decrease in the South-East Asian Region from 0.32% (0.18–0.47%) to 0.21% (0.10–0.32%).

**Table 1:** Estimated maternal and congenital syphilis and ABO cases and associated ANC service coverages, by WHO region. Annual country estimates of maternal syphilis prevalence, service coverage and CS case rates were weighted by the number of pregnancies in the country to generate regional and global estimates.

Region	Year	Pregnancies	Maternal syphilis prevalence	Pregnant women with active syphilis	ANC1 coverage	Syphilis screening coverage	Treatment coverage, mothers in ANC	Estimated ABO cases	Estimated non-clinical / non-ABO CS cases	Estimated total CS cases	Estimated CS case rate / 100,000 live births
**African region**	**2012**	35,055,000	1.62%	569,000	80%	35%	69%	247,000	222,000	469,000	1,377
	**2016**	37,150,000	1.52%	564,000	83%	47%	76%	216,000	188,000	404,000	1,119
**Region of the Americas**	**2012**	15,364,000	0.64%	98,000	97%	74%	84%	26,000	21,000	47,000	307
	**2016**	15,253,000	0.86%	131,000	97%	82%	88%	30,000	21,000	51,000	339
**Eastern Mediterranean Region**	**2012**	17,866,000	0.69%	124,000	78%	44%	82%	55,000	49,000	104,000	597
	**2016**	18,251,000	0.77%	140,000	78%	53%	85%	60,000	53,000	113,000	635
**European Region**	**2012**	11,449,000	0.11%	13,000	97%	90%	90%	1,930	1,470	3,400	30
	**2016**	11,289,000	0.10%	11,000	97%	94%	94%	1,260	940	2,200	19
**South-East Asia Region**	**2012**	37,889,000	0.32%	122,000	77%	59%	69%	46,000	39,000	85,000	231
	**2016**	36,987,000	0.21%	78,000	87%	65%	71%	28,000	25,000	53,000	145
**Western Pacific Region**	**2012**	24,802,000	0.28%	70,000	94%	81%	67%	22,000	19,000	41,000	165
	**2016**	24,297,000	0.26%	64,000	96%	84%	72%	20,000	18,000	38,000	156
**World**	**2012**	**142,425,000**	**0.70%**	**996,000**	**85%**	**59%**	**74%**	**397,000**	**352,000**	**749,000**	**540**
	**2016**	**143,227,000**	**0.69%**	**988,000**	**88%**	**66%**	**78%**	**355,000**	**306,000**	**661,000**	**473**

Between 2012 and 2016, the total number of pregnancies in the world increased from 142.4 million to 143.2 million. This increase combined with the stable maternal syphilis prevalence resulted in a slight decrease in the number of pregnant women with active syphilis: from 1.00 (0.89–1.23) million in 2012 to 0.99 (0.81–1.41) million in 2016 (Table 1).

### Service coverage

National data on ANC1 coverage, ANC-based syphilis testing coverage and syphilis treatment coverage from 2011 or later were identified for 182, 135 and 121 countries covering 65%, 76% and 75% of global pregnancies in 2016. Overall, 103 countries had data for all three indicators. Between 2012 and 2016 we estimated that global ANC1 attendance improved from 85% to 88%, syphilis testing coverage from 59% to 66% and treatment coverage from 74% to 78%. At the regional level, coverage rates improved or were stable across all regions for all three indicators. Only one region, the European Region, very nearly achieved the median regional service coverage rates required by WHO to achieve EMTCT (all three indicators above 95%), with 97% ANC1 attendance and 94% coverage of both syphilis screening and treatment in ANC.

### Congenital syphilis and adverse birth outcomes

The estimated total number of CS cases globally fell from 748,000 (684,000–812,000) in 2012 to 661,000 (538,000–784,000) in 2016, and the CS cases per 100,000 live births fell from 539 (493–585) to 473 (385–571; Table 1). Over the same period ABOs fell from 397,000 (361,000–432,000) to 355,000 (290,000–419,000) and non-clinical CS cases fell from 351,000 (321,000–381,000) to 306,000 (249,000–363,000).

The majority of cases of CS were in the African Region—which accounted for 62% and 61% of total CS cases in 2012 and 2016, respectively (Table 1). In four of the regions the number of ABOs and non-clinical CS cases fell. The two exceptions were the Eastern Mediterranean Region and the Region of the Americas. The largest decline in terms of absolute numbers was in the African Region (65,000) but the greatest proportionate decline was in the South-East Asia Region (42% drop). The estimated number of CS cases fell in 133 of the 205 countries.

**Fig 1:**
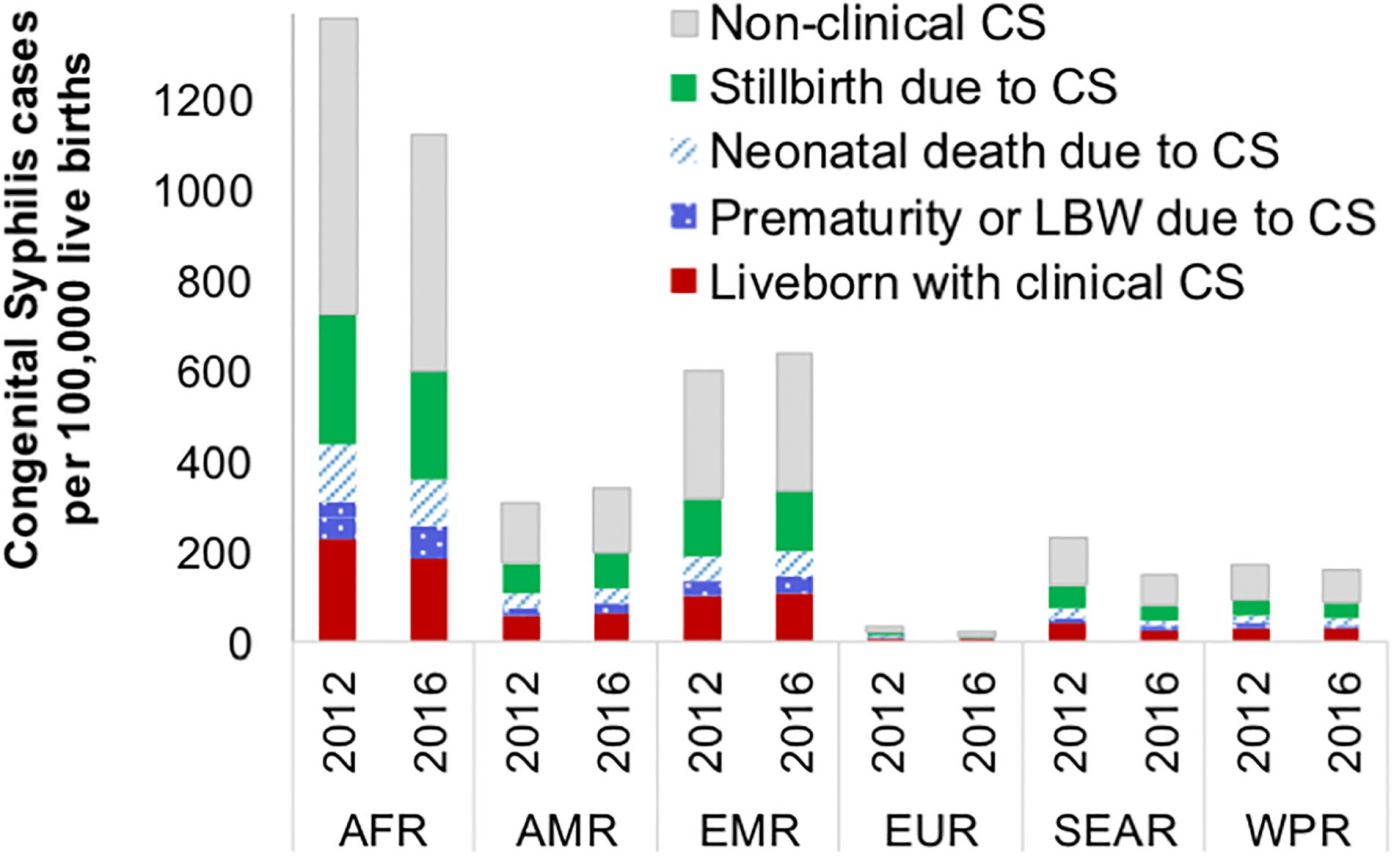
Distribution of CS case rates per 100,000 live births, by type, WHO region and calendar year. AFR = WHO African Region; AMR = WHO Region of the Americas; EMR = WHO Eastern Mediterranean Region; EUR = WHO European Region; SEAR = WHO South-East Asia Region; WPR = WHO Western Pacific Region.

The CS case rate was highest in the African Region (Table 1 and Fig 1), and lowest in the European Region, reflecting variations in the respective regions’ maternal prevalence and service coverages (Table 1). Across the 205 countries, in 2012 and 2016, 84 and 85 countries, respectively, had less than 50 CS cases per 100,000 live births, the WHO criterion for certifying EMTCT of syphilis. Countries with low CS case rates were mostly in the European and Western Pacific Regions; countries with high CS case rates in the African and Eastern Mediterranean Regions.

Restricting the analysis to the 100 countries that had a Spectrum-based maternal prevalence trend estimate as well as national data for all three ANC service coverage indicators, the estimated CS case rate in 2012 was 557 per 100,000 live births and 427 in 2016. This 23% decline is greater than that the CS case rate decline for all 205 countries (12%).

Early fetal deaths or stillbirths accounted for 143,000 of the estimated 355,000 global ABOs in 2016. Clinical disease in infants accounted for another 109,000, neonatal deaths for 14,000 and pre-maturity/LBW for 41,000 (Fig 1).

**Fig 2:**
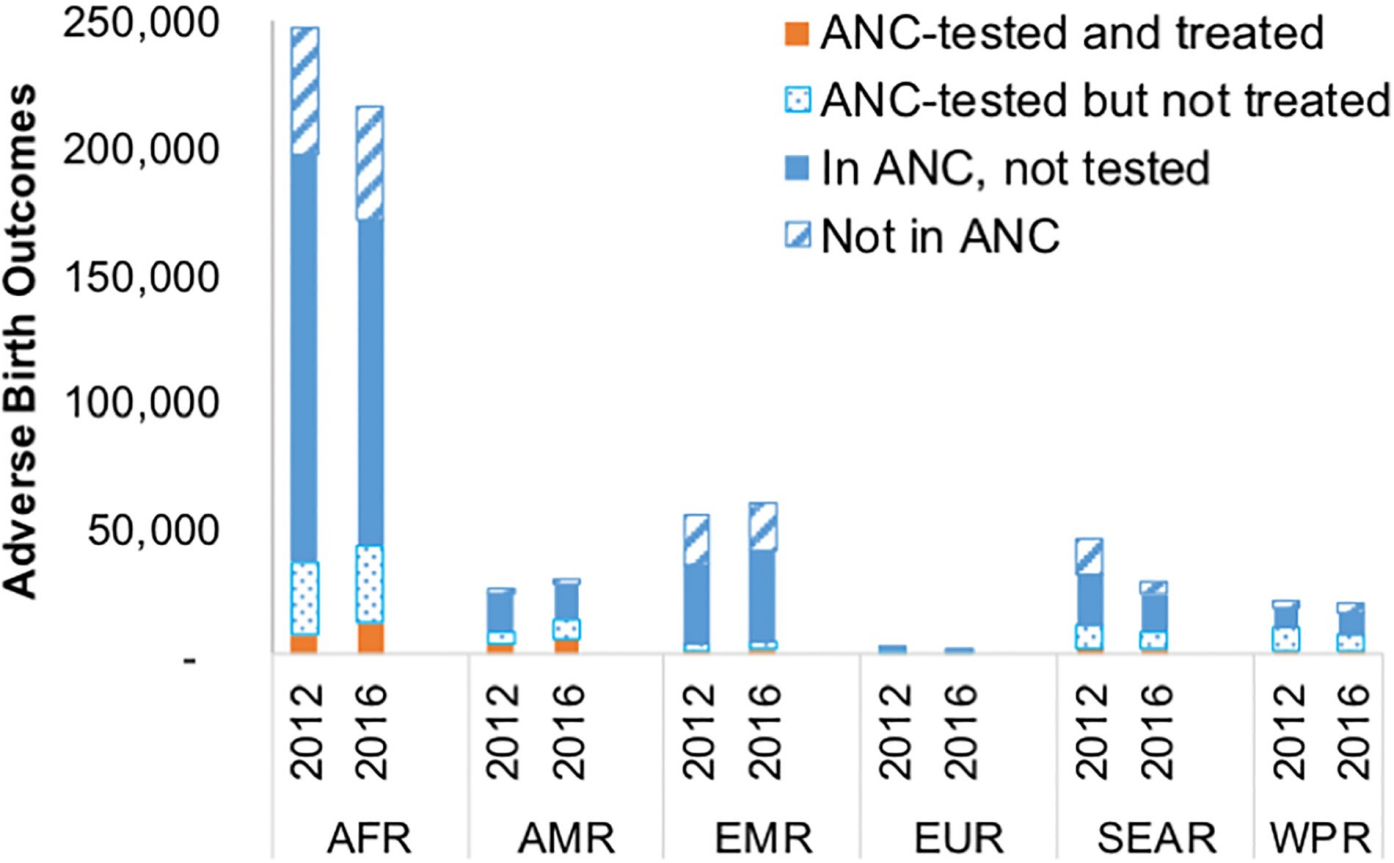
Estimated ABO cases in 2012 and 2016, by access to ANC care and treatment, WHO region and year. AFR = WHO African Region; AMR = WHO Region of the Americas; EMR = WHO Eastern Mediterranean Region; EUR = WHO European Region; SEAR = WHO South-East Asia Region; WPR = WHO Western Pacific Region.

Of the estimated global ABO cases in 2016, 74,000 (21%) were in mothers not enrolled in ANC, 203,000 (57%) in mothers who attended ANC but were not screened, 55,000 (16%) in mothers who had been screened but not treated and 23,000 (6%) in women screened and treated (due to treatment failures or reinfection during pregnancy). Comparable figures for 2012 were 89,000 (22%), 237,000 (60%), 55,000 (14%) and 16,000 (4%; Fig 2). The 42,000 drop in the number of ABOs between 2016 and 2012 was a composite effect of a drop in ABO cases among both women not enrolled in ANC (14,500) and in women who attended ANC but were not screened or screened but not treated (34,000), and an increase in ABOs occurring to women who had been treated (6,500) reflecting improvements in screening and treatment coverage, especially in the African Region.

### Sensitivity analysis

We conducted a series of univariate sensitivity analyses, focusing on the sensitivity of results to assumptions and imputations made for countries and indicators with limited or missing data (Table 2).

**Table 2:** Analyses of the sensitivity of estimates to uncertainties in country data.

Scenario	Maternal prevalence		CS case rate		CS case number: total		ABO number: total	
	2012	2016	2012	2016	2012	2016	2012	2016
Best estimate	0.70%	0.69%	539	473	748,000	661,000	397,000	355,000
Limit analysis to 100 countries with data for maternal prevalence and all 3 ANC coverage indicators	0.76%	0.66%	558	427	503,000	383,000		
Move Group B & D countries (time-constant maternal prevalence on national data) into Group E (imputing regional, time-varying maternal prevalence)	0.71%	0.69%	543	477	755,000	666,000	400,000	358,000
Replace imputed ANC1 coverage by the worldwide *lowest* ANC1 coverage, at each of 2012 and 2016*	Unchanged	Unchanged	541	475	752,000	664,000	398,000	357,000
Replace imputed ANC1 coverage by the worldwide *highest* ANC1 coverage, at each of 2012 and 2016**	Unchanged	Unchanged	538	473	748,000	661,000	396,000	355,000
Replace imputed Screening coverage by the worldwide *lowest* screening coverage, at each of 2012 and 2016*	Unchanged	Unchanged	569	508	791,000	709,000	418,000	379,000
Replace imputed Screening coverage by the worldwide *highest* Screening coverage, at each of 2012 and 2016**	Unchanged	Unchanged	508	445	706,000	621,000	376,000	336,000
Replace imputed maternal syphilis treatment coverage by the worldwide *lowest* coverage, at each of 2012 and 2016*	Unchanged	Unchanged	574	523	798,000	730,000	421,000	389,000
Replace imputed maternal syphilis treatment coverage by the worldwide *highest* coverage, at each of 2012 and 2016**	Unchanged	Unchanged	528	461	734,000	645,000	390,000	347,000

Notes to Table 2:

* Worldwide lowest coverage for ANC-1 attendance was 33.9% in 2012 and 40% in 2016; for screening coverage 0% and 6.3%, and for treatment coverage 9.2% and 26%.

** Worldwide highest coverage was 100% for ANC-1 attendance, screening and treatment, in both 2012 and 2016.

Restricting the analysis to the 100 countries with a Spectrum-based maternal prevalence trend estimate and national data for all three ANC service coverage indicators the estimated CS case rate fell from 557 per 100,000 live births in 2012 to 427 in 2016. This decline is greater than the decline for the complete data set of 205 countries (23% vs 12%).

Changing the maternal prevalence estimation approach for countries in Groups B & D from time-constant based on national data), to time-varying based on regionally imputed trends resulted in a negligible increase in maternal prevalence and resulting CS and ABO numbers.

In the best estimates, countries missing service coverage data were assigned the regionally imputed value for that indicator and year. For ANC-1 coverage replacing the imputed data by the lowest ANC-1 coverage reported by any country for that year led to a negligible increase in CS and ABO cases and rates and replacing them by the highest reported value resulted in a small decrease in CS and ABO cases.

Replacing screening coverage or treatment coverage with the lowest or highest recorded values resulted in more marked changes to CS and ABO estimates. In all cases, however, the estimated global decline in CS case rates and numbers was maintained; across all analyses this ranged from 9% to 24% for both the CS rate and number.

## Discussion

This analysis estimated a persistently high congenital disease burden resulting from maternal syphilis infection. Our results suggest that rates of CS and ABOs declined slightly (by 12%) from 2012 to 2016, reflecting continued progress towards CS elimination, but still some distance from the WHO target of 50 CS cases per 100,000 live births and at least 95% coverage in ANC1, syphilis screening and treatment [8]. CS case rates remain particularly high in the African and Eastern Mediterranean Regions, challenging elimination due to high maternal prevalence and low coverage of ANC services. For the South-East Asia Region, estimates for 2016 were considerably (though non-significantly) lower than for 2012, reflecting a decline in maternal prevalence; the European Region was the only region that (very nearly) reached the elimination threshold of ≤50 CS cases per 100,000 live births. Rises in CS cases in the Region of the Americas and the Eastern Mediterranean reflect the estimated increase in maternal prevalence. In the Americas, this parallels rising syphilis rates in the general population and among key HIV/STI risk groups such as men who have sex with men [17] which was not fully compensated by improving coverage in ANC-based screening and treatment. In the Eastern Mediterranean Region, data on both maternal syphilis prevalence and ANC-based coverage were relatively incomplete with a number of the large countries having no data, and hence the estimates for this region should be interpreted with caution.

In all regions, coverage of pregnant women attending ANC and ANC-based syphilis testing increased, with an increasing number of countries having introduced universal screening during pregnancy and integrated prevention of MTCT of HIV and syphilis into maternal health programs. Most ABOs were in women who attended ANC, suggesting that these outcomes could have been prevented had testing and treatment been done: in 2016, 88% of pregnant women globally had at least one ANC visit but among syphilis-infected mothers only 51% (= 66% test coverage, multiplied with 78% treatment coverage) of those who attended ANC were adequately treated; mothers attending ANC but not diagnosed or diagnosed but not treated accounted for 74% of the global CS burden.

The fact that treatment coverage is not 100% highlights the need to reduce patient loss-to-follow-up, by using point-of-care testing and same-day treatment within ANC, which has proven effective in reducing CS in some settings where loss to follow-up for test results and treatment was a problem [41]. Point-of-care tests are particularly beneficial in settings where women come to ANC later in pregnancy (after first trimester), because they limit delays in treatment that can result in treatment failures. A number of countries now use point-of-care rapid tests to diagnose maternal syphilis[42–44], and testing coverage may also improve as countries scale up the use of rapid dual HIV/syphilis tests, one of which one has received WHO pre-qualification [45]. Additionally, some countries using rapid testing still have government policies where only specialized STI centres can treat syphilis, upon confirmatory diagnosis, resulting in unnecessary loss to follow-up of infected women during this referral [46–48]. Stock-outs of both rapid tests and of benzathine-penicillin continue to challenge services in several countries; in addition to providers’ incorrectly prescribing penicillin substitutes due to misperceptions of quality or of the likelihood of adverse outcomes [49].

The revised 2012 estimate of 397,000 ABOs is higher than the previous 2012 estimate of 351,000 [15]. This reflects a slightly larger estimated number of pregnancies with active syphilis infection, and lower revised estimates of ANC-based testing coverage and maternal treatment coverage for 2012 (S3 File). We believe that the refinements made to the methods, and the increase in data availability, have substantially improved the quality of the 2012 estimates (S3 File). In particular, using the Spectrum-STI model and data extracted from the Spectrum-STI adult prevalence database [20–22], rather than relying on single-year values, to estimate maternal syphilis provided more stable national prevalence trends [17].

The key challenge with this type of analysis is the quality and quantity of input data, which in turn depend upon the quality and comprehensiveness of surveillance and health information systems. Challenges in estimating adult and maternal syphilis prevalence trends include the representativeness of ANC survey sites, representativeness of routine ANC-based services, and lack of recent ANC or population-based data in some countries (see [17,20] for a more detailed discussion). For 34 countries, a maternal prevalence trend could only be estimated by complementing ANC and general population data with blood donor measurements. In these countries there was no systematic difference in prevalence between the populations sampled; the median ratio of the prevalence in ANC and general population studies to blood donors was 1.07. However, in individual countries the ratio varied widely (95% range 0.0034–40), highlighting the need to improve our understanding of prevalence rates and drivers in different populations.

For the CS and ABOs estimates, service coverage data were missing for a number of countries and in other countries the data may have been incorrect. Seventy countries (covering 24% of pregnancies in 2016) lacked national data on the coverage of syphilis testing coverage, and 85 (covering 25% of global pregnancies in 2016) lacked treatment coverage data and for these countries the regional mean was imputed. In addition, there were a number of countries reporting 100% screening and/or treatment coverage. For some countries the reported universal coverage may have been real but for many it was not and probably based on a non-representative subset of the population. In the absence of data, we were not able to adjust the numbers and, as a result, for this subset of countries our CS and ABO estimates are probably too low. Also, larger decline in CS and ABOs in the 100 countries with complete data, compared to all 205 countries, suggests that imputing values for countries may have masked some of the progress towards the global EMTCT targets. On the other hand, countries with more and better data may have better services and coverage, which may have caused over-estimation of progress. The sensitivity analyses suggested some uncertainty in burden and extent of the decline at the national level. At the global level the uncertainties were smaller (Table 2) as most of the larger, most fertile and higher STI burden countries had the required data (S2 File). Key to improving future estimates will be expanding and strengthening data collection and reporting. These data are also crucial for monitoring and programme planning and will be essential if countries want to be validated by WHO as having eliminated mother-to-child transmission of syphilis. More complete country data may also enable statistical assessment of time trends.

The estimates did not take into account changes over time at the country level in timing of first ANC visit [50]. Among 11 countries with at least two surveys that measured the proportion of pregnancies with an early ANC visit between 2012 and 2016 the average time of first ANC visit improved slightly (from a median of 14.4 weeks in the first survey to 14.0 weeks in the second survey). By ignoring the progress with early ANC enrolment, we may have slightly overestimated the 2016 burden and under-estimated the decline in CS and ABO from 2012 to 2016. This would especially affect countries with high coverage of ANC1, syphilis testing and treatment. Similarly, we did not account for possible increases in the proportion of ANC women screened more than once during the pregnancy, thus reducing missed diagnoses due to re-infection. Nevertheless, neither of these simplifications greatly affected the current global estimates, which in 2016 continue to be driven by outcomes for women not diagnosed and treated for their syphilis at any time during the pregnancy.

In conclusion, the new global estimates of maternal and congenital syphilis indicate continued progress towards the elimination of mother-to-child transmission of syphilis. Untreated syphilis, however, continues to result in substantial numbers of adverse birth outcomes, most of which occurred among women who were enrolled in ANC but did not receive recommended services for CS prevention. Reducing preventable infant deaths will require accelerating the ongoing improvements in access to ANC care, ensuring that all women attending ANC care are screened for syphilis early in pregnancy and treated appropriately, and expanding efforts to test and treat partners and populations at higher risk in order to lower overall community prevalence levels. In countries with low screening and treatment coverage, or where pregnant women come to ANC late in pregnancy, the use of rapid syphilis tests and treatment with benzathine penicillin at the same clinic visit could accelerate declines in CS. Additionally, controlling syphilis in the general population, including improved management of partners of mothers diagnosed during ANC, will be key to eliminate congenital syphilis as a public health problem by 2030.

## Supporting information

S1 File ABO risk probabilities in treated women. (DOCX)

S2 File Country estimates of maternal syphilis prevalence and congenital syphilis incidence and their ANC-related service coverage determinants. (XLSX)

S3 File Comparison of WHO 2008, 2012 and 2016 estimates of ABOs. (DOCX)

## Abbreviations

ABO: Adverse Birth Outcome
ANC: antenatal clinic
ANC1: attendance in antenatal care at least once during a pregnancy
CI: confidence interval
CS: Congenital Syphilis
EMTCT: (elimination of) mother-to-child transmission
LBW: Low Birth Weight
N: sample size
RPR: Rapid Plasma Reagin test
STI: sexually transmitted infection
TPHA: *Treponema pallidum* hemagglutination assay
WHO: World Health Organization
