# Correction: Global burden of maternal and congenital syphilis and associated adverse birth outcomes—Estimates for 2016 and progress since 2012

**DOI:** 10.1371/journal.pone.0219613

**Published:** 2019-07-05

**Authors:** Eline L. Korenromp, Jane Rowley, Monica Alonso, Maeve B. Mello, N. Saman Wijesooriya, S. Guy Mahiané, Naoko Ishikawa, Linh-Vi Le, Morkor Newman-Owiredu, Nico Nagelkerke, Lori Newman, Mary Kamb, Nathalie Broutet, Melanie M. Taylor

In the Congenital syphilis and adverse birth outcomes subsection of the Results, there is an error in the second sentence of the fifth paragraph. The correct sentence is: Clinical disease in infants accounted for another 109,000, neonatal deaths for 61,000 and pre-maturity/LBW for 41,000 (Fig 1).

## References

[1] Korenromp EL, Rowley J, Alonso M, Mello MB, Wijesooriya NS, Mahiané SG, et al (2019) Global burden of maternal and congenital syphilis and associated adverse birth outcomes—Estimates for 2016 and progress since 2012. PLoS ONE 14(2): e0211720 10.1371/journal.pone.0211720 30811406 PMC6392238

