# Supplementary material for: Global burden of maternal and congenital syphilis and associated adverse birth outcomes—Estimates for 2016 and progress since 2012
Source: PLoS One. 2019 Feb 27;14(2):e0211720. doi: 10.1371/journal.pone.0211720 (PMC6392238; doi:10.1371/journal.pone.0211720)
Supplement: S1 File — (DOCX) [file pone.0211720.s001.docx]

# Supporting Information file 1

Of the study ‘*Global burden of maternal and congenital syphilis and associated adverse birth outcomes – estimates for 2016 with evaluation of progress since 2012’* , by Korenromp-EL, Rowley-J et al.

Version 23 January 2019

## S1. ABO risk probabilities in treated women

In previous WHO estimations, the global risk of a syphilis-attributable ABO in mothers treated for syphilis was assumed to be independent of the timing of treatment during the pregnancy. Data from a number of studies, however, suggest that maternal treatment is more effective the earlier a woman is treated during a pregnancy (1, 2). Thus, countries with a pattern of early ANC enrolment and syphilis testing and treatment would be expected to have lower CS and ABO rates than countries with a pattern of late ANC enrolment and syphilis testing and treatment. This effect may well contribute to the wide variations observed in routinely reported CS case rates between countries with different levels of ANC service coverage.

The ABO risk probability to mothers treated for syphilis during ANC used the timing of first ANC visit as a proxy for the time of the syphilis treatment, and combined this with a gradient of ABO risk for syphilis-treated mothers that reflected timing of treatment.

### Timing of first ANC visit and maternal syphilis treatment

The timing of maternal syphilis testing and treatment during ANC is not a routinely collected indicator. However, data the proportion of pregnancies with early enrolment in ANC visits are available for 133 countries from 216 periodic national demographic and health surveys conducted in between 1985 and 2015 (median year, 2004) collated in a WHO database (<http://www.who.int/reproductivehealth/early-anc-estimates/en/>). Since the definition of early enrolment in these 216 surveys varied across countries and in some countries over time, we calculated for each survey that had measured the proportion of early ANC enrolment, the average timing (as, weeks in the pregnancy) of first ANC visit, using the interpretation shown in Table S1.1.

The distribution of ANC1 ranged from 10.9 weeks (in the UK in 2010) to 26.7 weeks (in Tunisia in a survey covering pregnancies over 1989-1995), with a median of 17.8 weeks and average of 17.9 weeks. Imputed regional medians from surveys conducted in 2010 or later were: 20.6 weeks in the African Region, 17.0 weeks in the Region of the Americas, 17.3 weeks in the Eastern Mediterranean Region, 13.9 weeks in the European Region, 19.5 weeks in the South-East Asia Region and 16.6 weeks in the Western Pacific Region.

Country-specific time of ANC1 for all countries were then generated, taking the most recent survey for countries which had had two measurements. For countries without a survey, the WHO regional median was imputed from surveys conducted in 2010 or later.

**Table S1.1 Estimated average time of first ANC visit, and corresponding assumed ABO risk probability for syphilis-treated mothers averaged over all pregnancies in the country**

| Cut-off timing (weeks in the pregnancy) to define early ANC | **Average weeks in the pregnancy, of first ANC** | | **ABO risk probability / untreated syphilis-infected mother** | |
| --- | --- | --- | --- | --- |
| ***Group of ANC women, by timing of first visit:*** | ***Early*** | ***Late*** | ***Early*** | ***Late*** |
| <12 weeks | 10 | 20 | 2.0% | 5.0% |
| <13 weeks | 11 | 21 | 2.3% | 5.5% |
| <14 weeks | 12 | 22 | 2.5% | 6.0% |
| <3 months | 12 | 22 | 2.5% | 6.0% |
| 1st trimester | 12 | 22 | 2.5% | 6.0% |
| <15 weeks | 13 | 23 | 2.6% | 6.5% |
| <16 weeks | 14 | 24 | 2.7% | 7.5% |
| <4 months | 15 | 24 | 3.0% | 8.5% |
| <20 weeks | 17 | 26 | 3.5% | 11.0% |
| <6 months | 20 | 28 | 5.0% | 14.0% |

### ABO risk probability in treated women

The ABO risk probability in treated women was assumed to depend on the timing of syphilis treatment during the pregnancy. A curve of ABO risks was fitted (indicating a country’s average across all pregnancies) that increased exponentially with later timing of first ANC, the pattern established in meta-analyses by Qin et al. (1) and Hawkes et al. (2)).

In the fitted curve (Figure S1.1), the ABO risk is below the value used in previous WHO estimates (fixed at 8.2%) ((3, 4). The latter had been based on a meta-analysis of ABO risks to untreated mothers in 6 studies in 5 countries (5), combined with estimates of the effectiveness of treatment (at any time during the pregnancy) in reducing that risk (6) from meta-analysis of between 3 and 8 studies (the number varying by outcome) in selected countries. In contrast, our country-stratified ABO risk assumption (Figure S1.1) approached 0 for very early ANC enrolment, fitting with low CS case reports in some countries that qualified for WHO certification of CS elimination, where CS case reports are believed to be reasonably complete given their near-universal coverage of (early) ANC (7, 8).

Our fitted ABO risk curve corresponded to an odds ratio of ABO between women with ANC1 in the third versus the first trimester of pregnancy of 5.0. This is similar to the 4.9 odds ratio (64% relative to 13%) in the Qin et al. meta-analysis (1) and higher than the 2.24 odds ratio found in the Hawkes et al. meta-analysis of a subset of the same studies (2).

Applying the ABO risk gradient to the 216 country-years with data on the timing of ANC1, the median country-specific ABO risk was 4.5%, the average was 5.0% and the country values ranged from 2.3% (UK 2010 survey) to 12.5% (Tunisia 1989-1995 survey) (Figure S1.1A).

The corresponding regional median ABO risks were extrapolated from the subset of surveys with data from in 2010 or later (Figure S1.1B), and these were used for countries without a survey of timing of ANC1. The regional medians were: 6.8% (range, 3.7-10.4%) in the African Region, 4.4% (2.7-6.8%) in the Region of the Americas, 5.5% 3.5-12.5%) in the Eastern Mediterranean Region, 3.4% (2.3-6.0%) in the European Region, 6.0% (2.9-7.9%) in the South-East Asia Region and 4.7% (2.3-12.2%) in the Western Pacific Region.

**Figure S1.1 Distribution of timing of first ANC visit, and corresponding ABO risk probabilities assumed as input to national ABO estimates:**

(A) for 216 surveys i.e. country-years that measured early ANC enrolment for pregnancies, conducted over 1990-2015, covering pregnancies over 1985 to 2015; (B) for a subset of surveys conducted within 2010-2015, covering pregnancies over 2005 to 2015.

1. (B)

Note to Figure S1.1: Blue dots represent ABO risk assumptions for given average times of first ANC as measured in surveys; the red dashed lines represent the ABO risk assumption in the WHO 2008 and 2012 CS estimates.

### Alternative global CS and ABO estimates, varying the assumed ABO risk to treated mothers

Finally, we assessed the sensitivity of global CS and ABO results, to the assumed ABO risk remaining for mothers treated after ANC-based testing. This assumption made little difference to the estimated global CS and ABO numbers and rates, as the assumption only applied to the subset of syphilis-infected mothers who were diagnosed and treated, and mainly so in lower-syphilis-burden countries (Table S1.2).

**Table S1.2 Global CS and ABO estimates, 2012 and 2016 under alternative assumptions of the ABO risk to syphilis-infected, treated mothers**

| **Scenario of ABO risk to syphilis-infected, treated mothers** | **CS case rate: average*** | | **CS case number: total** | | **ABO number: total** | |
| --- | --- | --- | --- | --- | --- | --- |
|  | **2012** | **2016** | **2012** | **2016** | **2012** | **2016** |
| **Best estimate: gradient, depending on countries’ timing of first ANC visit** (global average*: 5.1%) | **539** | **473** | **748,000** | **661,000** | **397,000** | **355,000** |
| ABO risk for treated mothers: 0% | 527 | 456 | 731,000 | 638,000 | 380,000 | 332,000 |
| ABO risk for treated mothers: 8% fixed (as in WHO 2008 and 2012 estimations) | 543 | 477 | 753,000 | 667,000 | 402,000 | 361,000 |

Footnotes to Table S1.2: Totals and averages for 205 countries.

* Pregnancies-weighted average.

## References of the Supporting file 1

1. Qin J, Yang T, Xiao S, Tan H, Feng T, Fu H. Reported estimates of adverse pregnancy outcomes among women with and without syphilis: a systematic review and meta-analysis. *PLoS One*. 2014;**9**(7):e102203. 10.1371/journal.pone.0102203, PONE-D-14-04391 [pii].

2. Hawkes SJ, Gomez GB, Broutet N. Early antenatal care: does it make a difference to outcomes of pregnancy associated with syphilis? A systematic review and meta-analysis. *PLoS One*. 2013;**8**(2):e56713. 10.1371/journal.pone.0056713, PONE-D-12-31492 [pii]

3. Wijesooriya NS, Rochat RW, Kamb ML, Turlapati P, Temmerman M, Broutet N, Newman L. Global burden of maternal and congenital syphilis in 2008 and 2012: a health systems modelling study. *Lancet Glob Health*. 2016;**4**(8):e525-33. 10.1016/S2214-109X(16)30135-8

4. Newman L, Kamb M, Hawkes S, Gomez G, Say L, Seuc A, Broutet N. Global estimates of syphilis in pregnancy and associated adverse outcomes: analysis of multinational antenatal surveillance data. *PLoS Med*. 2013;**10**(2):e1001396. 10.1371/journal.pmed.1001396

5. Gomez GB, Kamb ML, Newman LM, Mark J, Broutet N, Hawkes SJ. Untreated maternal syphilis and adverse outcomes of pregnancy: a systematic review and meta-analysis. *Bull World Health Organ*. 2013 Mar 01;**91**(3):217-26. 10.2471/BLT.12.107623, BLT.12.107623 [pii]

6. Blencowe H, Cousens S, Kamb M, Berman S, Lawn JE. Lives Saved Tool supplement detection and treatment of syphilis in pregnancy to reduce syphilis related stillbirths and neonatal mortality. *BMC Public Health*. 2011 Apr 13;**11 Suppl 3**:S9. 10.1186/1471-2458-11-S3-S9, 1471-2458-11-S3-S9 [pii]

7. Pan-American Health Organization, UNICEF. Elimination of mother-to-child transmission of HIV and syphilis in the Americas -- update 2016. Washington DC 2016. <http://iris.paho.org/xmlui/bitstream/handle/123456789/34072/9789275119556-eng.pdf>

8. UNICEF East Asia and Pacific Regional Office, World Health Organization Western Pacific regional office, UNAIDS. Elimination of Parent-to-Child Transmission of HIV and Syphilis in Asia and the Pacific in 2015 and Beyond Progress Review and Roadmap. Bangkok 2016 August. <http://www.wpro.who.int/hiv/documents/topics/pmtct/20160909-eptct-progress-report.pdf?ua=1>
