# Supplementary material for: Global burden of maternal and congenital syphilis and associated adverse birth outcomes—Estimates for 2016 and progress since 2012
Source: PLoS One. 2019 Feb 27;14(2):e0211720. doi: 10.1371/journal.pone.0211720 (PMC6392238; doi:10.1371/journal.pone.0211720)
Supplement: S3 File — (DOCX) [file pone.0211720.s003.docx]

# Supporting Information file 3

Of the study ‘*Global burden of maternal and congenital syphilis and associated adverse birth outcomes – estimates for 2016 with evaluation of progress since 2012’* , by Korenromp-EL, Rowley-J et al.

Version 23 January 2019

## Comparison of WHO 2008, 2012 and 2016 estimates of ABOs

Table S3.1 summarizes ABO numbers from the 2008, 2012 and 2016 estimation rounds. Whilst the methods were similar in the three studies, there were some changes made that mean they are not directly comparable. We were not able to compare the corresponding number of total CS cases (i.e., ABOs plus non-clinical CS cases), as the 2008 and 2012 exercises had not estimated this outcome.

**Table S3.1 Numbers of ABOs estimated in three rounds of WHO estimations**

Notes to Table S3.1: * Pregnancies-weighted.

** This study did not provide global estimates of treatment coverage, but the country data inputted into the calculation would correspond to over 100% global treatment coverage in 2008.

Number of countries and territories: The increase from 193 in 2008 to 194 countries for the 2012 estimates (1) reflects the split of Sudan into Sudan and South Sudan. For the 2016 estimates, we added 11 countries and territories: Aruba, Cayman Islands, French Guiana, Guadeloupe, Martinique, Montserrat, Netherlands Antilles, Puerto Rico, Turks and Caicos Islands, State of Palestine and Kosovo.

Pregnancies: The increased number of pregnancies between the previous and current 2012 estimations reflects a demographic update in the World Population Projections (2). The 11 countries and territories added in our current estimations accounted for less than 1% of global pregnancies (in 2012), and hence did not significantly influence the global level results.

Maternal syphilis prevalence: The new estimates (2012 and 2016) are based on an improved dataset, compared to the earlier 2008 and 2012 WHO estimates (1, 3). Annual estimates were based on a national time trend estimation for 167 countries, rather than a single prevalence data point. The number of countries where the data were imputed fell from 72 to 24 (Table S3.2).

Our projections of historic trends in maternal syphilis prevalence pre-2012 (4) suggest that the 2008-2012 declines in maternal prevalence estimated were overestimated, as our 2008 pregnancy-weighted 2008 syphilis prevalence estimate is 0.91% (0.84-0.98%) and not 1.33% as before (1). This change reflects the expanded prevalence data set now available, where observed prevalences over 2008-2012 are on average lower than in the dataset used in the previous round, possibly due to the expansion of syphilis surveillance (routine testing and sentinel surveys) and increasing coverage of lower-prevalence areas, as observed also in ANC-based HIV surveillance (5).

Service coverage indicators: The revised 2012 estimates are based on an improved data set with fewer imputations (Table S3.2). The new figures suggest a slightly higher coverage of ANC1 in 2012, but lower coverages of ANC-based syphilis screening and especially syphilis treatment, than had been assumed previously.

ABO cases: We estimated a larger number of ABOs for the year 2012, based on a larger number of pregnancies (143 million instead of 138 million) and lower estimated treatment coverage among ANC-diagnosed mothers (78% instead of 85%). In the 2008 (3) and 2012 (1) estimations, as well as in our current re-estimation for 2012, on average 40% of syphilis-infected pregnancies (e.g. 397,000 out of 1.00 million) resulted in an ABO. We estimated this to have fallen to 36% by 2016, reflecting the progressive impact of testing and (early) treatment.

The current estimates suggest a relatively small recent decline in ABO rates over 2012-2016. A large decline in ABOs from 2008 to 2012 was previously estimated driven by a strong decrease in maternal syphilis prevalence and despite little improvement in ANC service coverages over that period (Table S3.1). The (re-)estimations, in contrast, suggest a stable maternal prevalence over 2012-2016, yet some decreases in ABO numbers thanks to improving coverage of ANC1 attendance, ANC-based testing and maternal treatment.

**Table S3.2 Data that formed the basis of the three sets of WHO estimates**

## References of the Supporting file 3

1. Wijesooriya NS, Rochat RW, Kamb ML, Turlapati P, Temmerman M, Broutet N, et al. Global burden of maternal and congenital syphilis in 2008 and 2012: a health systems modelling study. Lancet Glob Health. 2016;4(8):e525-33.

2. World Population Prospects 2017 [Internet]. 2017 [cited 16 November 2017]. Available from: <http://esa.un.org/unpd/wpp/>.

3. Newman L, Kamb M, Hawkes S, Gomez G, Say L, Seuc A, et al. Global estimates of syphilis in pregnancy and associated adverse outcomes: analysis of multinational antenatal surveillance data. PLoS Med. 2013;10(2):e1001396.

4. Korenromp EL, Mahiané SG, Nagelkerke N, Taylor M, Williams R, Chico RM, et al. Syphilis prevalence trends in adult women in 132 countries – estimations using the Spectrum Sexually Transmitted Infections model. Scientific Reports. 2018;8(1).

5. Montana LS, Mishra V, Hong R. Comparison of HIV prevalence estimates from antenatal care surveillance and population-based surveys in sub-Saharan Africa. Sex Transm Infect. 2008;84 Suppl 1:i78-i84.
